# Novel Predictive Biomarkers in the Head and Neck Squamous Cell Carcinoma (HNSCC)

**DOI:** 10.3390/jcm13195876

**Published:** 2024-10-02

**Authors:** Magdalena Miniuk, Joanna Reszeć-Giełażyn, Piotr Bortnik, Agata Borsukiewicz, Aleksandra Mroczek

**Affiliations:** 1 Department of Medical Pathomorphology, Medical University of Bialystok, 15-269 Bialystok, Poland; joanna.reszec-gielazyn@umb.edu.pl; 2 Department of Maxillofacial and Plastic Surgery, Medical University of Bialystok, 15-276 Bialystok, Poland; czaves82@wp.pl (P.B.);; 3 Department of Hygiene, Epidemiology and Metabolic Disorders, Medical University of Bialystok, 15-089 Bialystok, Poland; 40446@student.umb.edu.pl

**Keywords:** head and neck squamous cell carcinoma (HNSCC), immunotherapy, predictive biomarkers, PD-L1, PD-1, CTLA-4, p53, HPV

## Abstract

Head and neck squamous cell carcinoma (HNSCC) is the sixth most common cancer worldwide, characterized by high aggressiveness and frequent metastasis to regional lymph nodes. Despite advances in therapy, including checkpoint inhibitor immunotherapy, surgery, radiotherapy, and chemotherapy, survival rates for patients with advanced HNSCC remain unsatisfactory. This article presents the latest research on predictive biomarkers such as PD-L1, PD-1, CTLA-4, p53, and HPV, which may enhance treatment efficacy and improve clinical outcomes for patients. The clinical value of these biomarkers, their limitations, and their potential application in HNSCC therapy are emphasized. Special attention is given to immunotherapy, which shows promising results in treating this type of cancer through the modulation of the immune response. The review’s findings highlight the need for further research on new biomarkers to develop more personalized and effective therapeutic strategies for HNSCC patients.

## 1. Introduction

Head and neck squamous cell carcinoma (HNSCC) is currently the sixth most common cancer worldwide. According to 2022 data, there were recorded approximately 66,470 new cases of HNSCC and 15,050 deaths due to the disease. In Europe, HNSCC accounts for 4% of all adult cancers, occurring twice as often in men than in women. HNSCC is the most common malignant tumor found in the human oral cavity. According to the latest forecasts, the number of new HNSCC cases will increase by 40% over the next 16 years [1,2,3,4,5].

Recent studies have confirmed that this highly malignant disease arises from exposure to exogenous carcinogens such as well-known tobacco and alcohol, but also HPV viruses, chronic inflammation caused by pathogenic microflora, and the host’s genetic susceptibility. These factors can irreversibly damage the genetic material of epithelial cells and promote neoplastic transformation [6,7].

To determine effective treatment methods, it seems crucial to consider the aggressive course and characteristic invasion of regional lymph nodes in the neck and understand the mechanism of HNSCC metastases via the lymphatic. Growing evidence indicates that tumor development, invasion, and metastasis capability depend not only on deregulated biological responses within the cancer cell but also on the influence of the complex ecosystem surrounding the tumor cell (TME). To avoid the host’s immune attack, cancer cells must create an immunosuppressive environment that includes the disintegration of immune cells, dysfunction of inflammatory cytokines, and induction of immune checkpoints [6,8].

Humans are equipped with a complex defense mechanism against the threats posed by carcinogens, whose prevalence is constantly increasing. Cytotoxic T lymphocytes are considered the main defensive arsenal involved in the immune response against HNSCC. Their membranes contain immune checkpoints—immunosuppressive molecules (e.g., PD-1—programmed death protein, CTLA-4—cytotoxic T-lymphocyte-associated protein 4) that weaken T lymphocytes’ defensive functions and maintain the host’s tolerance to threats. Regrettably, tumor cells, acting like invisible enemies, have learned to exploit T-cells’ checkpoints by avoiding immune response [9].

**Figure 1:**
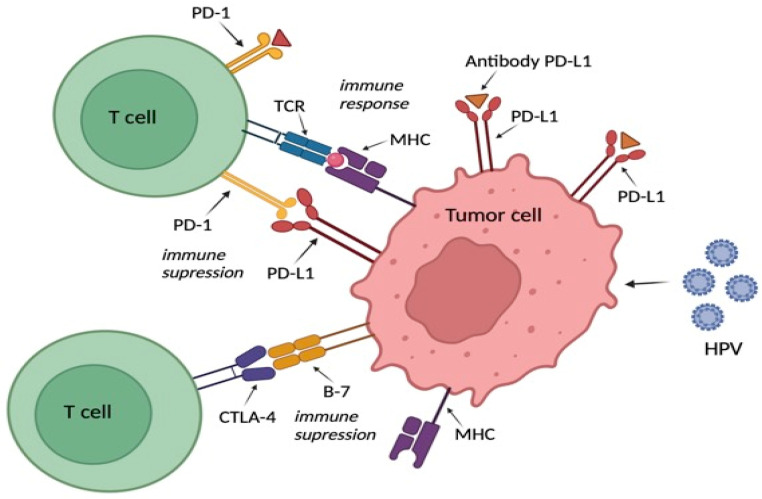
The role of PD-L1 and PD-1 connection, CTLA-4. Created with © 2024 BioRender.

PD-1 and its ligand (PD-L1) have garnered significant scientific interest due to their strong antitumor effects stemming from blocking the immunosuppressive pathway. Some immune checkpoint inhibitors (ICI) targeting the PD-1/PD-L1 axis have been developed to treat several human cancers [10]. Both CTLA-4 and PD-1, common inhibitory checkpoints observed on activated T cells, have been identified as the most reliable targets in cancer treatment. To date, six drugs targeting PD-1 or its ligand PD-L1 and one drug targeting CTLA-4 have been approved for treating various cancers, with several others in advanced stages of development [11] (Figure 1).

Despite the use of checkpoint inhibitor immunotherapy, improvements in surgical methods, the use of radio- and chemotherapy, and the combination of these methods, high mortality due to advanced HNSCC persists. In clinical practice, few biomarkers have been approved for routine use in therapy. Half of the patients with advanced HNSCC experience disease recurrence, and five-year survival rates remain unsatisfactory. This highlights the necessity of identifying additional predictive biomarkers to increase treatment effectiveness, prolong survival, and provide better disease control.

In this review, we will present the latest predictive biomarkers and discuss their clinical value, limitations, and potential use in treating patients with advanced HNSCC.

## 2. Methods

Our investigation in this review is based on publications available in the PubMed and Google Scholar scientific search engines. Additional research included materials from the meetings of the European Society for Medical Oncology (ESMO) and the American Society of Clinical Oncology (ASCO). Keywords used to search literature covered the period from January 2019 to December 2024 regardless of article type and were as follows: “predictive biomarkers in HNSCC, PD-L1 HNSCC, CTLA-4 HNSCC, p53 HNSCC, HPV HNSCC, immunotherapy in HNSCC”. Initially, duplicate articles, articles written in languages other than English, and paywalled articles were excluded. The remaining articles were then assessed based on their title, followed by their abstract. Articles that did not consider the predictive role of the described biomarkers were eliminated. All articles that met the following inclusion criteria were analyzed in full-text form. Ultimately, 47 manuscripts were included during the study.

Inclusion criteria: (1) Articles providing detailed information on predictive biomarkers, including their correlation with treatment and/or survival of HNSCC patients. (2) Articles in English. (3) Open-access articles (only fee-full texts).

Exclusion criteria: (1) Articles in languages other than English. (2) Paywalled or without open access articles. (3) Articles referencing the role of biomarkers in cancers other than HNSCC (nasopharyngeal, skin, etc.) (4) Articles that do not provide detailed information relevant to the objectives of this review.

### 2.1. PD-L1

The PD-1 receptor, also known as the “death receptor”, regulates immune responses by promoting apoptosis of T lymphocytes. It occurs due to binding of the death receptor ligand, PD-L1, observed on some cancer cells. This process allows cancer cells to evade the host’s immune response. Currently, many studies focus on evaluating therapies using immune checkpoint inhibitors (ICI). The application of immunotherapy in the treatment of oral cancers continues to evolve [12].

The KEYNOTE-048 study, a randomized, open-label phase III trial, showed that patients with higher PD-L1 CPS scores (20 or more and 1 or more) achieved better overall survival outcomes when treated with pembrolizumab monotherapy, compared to traditional chemotherapy with cetuximab. The association of high PD-L1 expression with better response to pembrolizumab highlights the significance of PD-L1 as a predictive biomarker. Thus, pembrolizumab in combination with platinum and 5-fluorouracil was approved for the treatment of R/M HNSCC regardless of PD-L1 status, while pembrolizumab monotherapy was approved as a first-line treatment for R/M HNSCC with positive PD-L1 [13].

Current results from the KEYNOTE-048 phase III trial, after a 4-year follow-up, confirm the benefits of treatment with pembrolizumab or pembrolizumab with chemotherapy regarding the survival of the patients [14].

**Figure 2:**
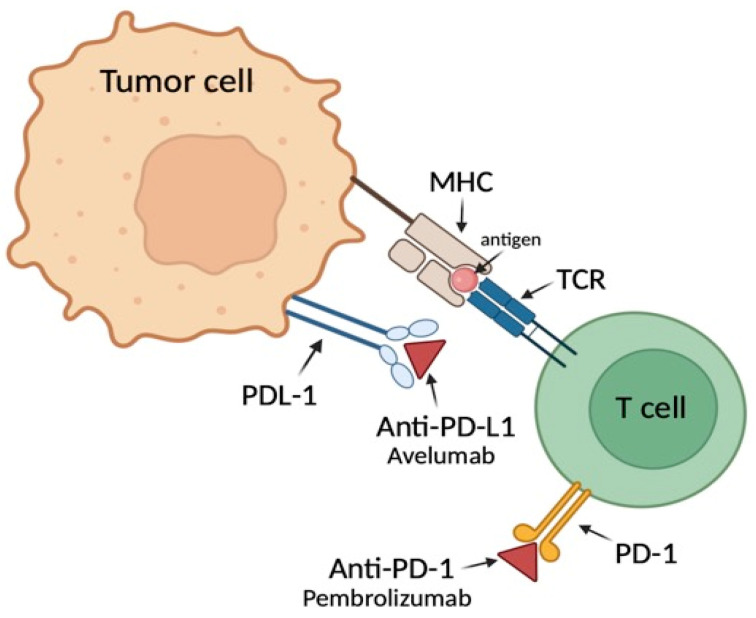
The mechanism of anti-PD-L1 and anti-PD-1 in treatment. Created with © 2024 BioRender.

Between 2014 and 2017, in the KEYNOTE-040 randomized phase III trial, the efficacy and safety of pembrolizumab therapy were compared with standard therapy (methotrexate, docetaxel, or cetuximab) in patients with recurrent or metastatic HNSCC. It was found that monotherapy with the checkpoint inhibitor, compared to standard therapy, extended overall survival (8.7 months for pembrolizumab, 7.1 months for standard therapy), and the benefits of ICI treatment were greater, the higher the expression of PD-L1 in the tumor was. Additionally, pembrolizumab treatment demonstrated lower toxicity and a better safety profile. The results underscore that PD-L1 expression level is a significant predictor of response to pembrolizumab. Patients with higher PD-L1 scores benefit more from the treatment than those with the lower PD-L1 scores [15] (Figure 2).

The CheckMate 141 randomized phase III trial confirmed the superiority of nivolumab therapy compared to chemotherapy in terms of overall survival in 361 patients with R/M HNSCC. The study was continued for another 2 years, and long-term analyses maintained the initial thesis [16]. Interestingly, in the CheckMate 141 update regarding the 2-year survival with PD-L1 analysis in the tumor, benefits from ICI therapy were found regardless of PD-L1 expression status and HPV status [17].

In the KEYNOTE-055 study evaluating the efficacy of pembrolizumab therapy in patients with R/M HNSCC priorly treated with cetuximab, it was found that 82% of tumors showed positive PD-L1 expression (based on combined CPS scoring), with an overall response rate of 18%, in comparison with 12% in PD-L1 negative tumors. Patients with positive PD-L1 results demonstrated higher overall survival after 6 months [12].

The effectiveness of avelumab-cetuximab-radiotherapy and standard care in patients with locally advanced HNSCC were compared in the randomized phase III GORTEC-REACH study. The first therapy (Cetux-SOC) included cetuximab and radiotherapy, while in the second (Exp) patients additionally received avelumab. The results showed that adding avelumab to standard therapies in the group of patients unsuitable for cisplatin treatment resulted in better progression-free survival (44% in the Exp group, 31% in the Cetux-SOC group), had a beneficial effect on local-regional control within 2 years (34% in Exp and 44% Cetux-SOC), reduced distant metastases, and improved long-term survival (58% in the Exp group and 54% in Cetux-SOC).

The “Nivolumab (N) + Ipilimumab (I) vs. EXTREME as first-line treatment for R/M SCCHN: the final result of CheckMate 651” compared the efficacy of nivolumab and ipilimumab therapy with the cetuximab-cisplatin/carboplatin and fluorouracil regimen in 947 patients with R/M HNSCC. The study did not show a statistically significant improvement in overall survival (OS) in patients treated with the N + I combination (nivolumab + ipilimumab) compared with the standard EXTREME regimen (cetuximab + cisplatin/carboplatin + fluorouracil). However, there were observed therapeutic benefits in patients treated with N + I with high PD-L1 levels (CPS 20) and in the CPS 1 group, manifesting as long-term overall survival (OS) and durable responses. The study also highlighted the favorable safety profile of N + I therapy compared to the standard EXTREME treatment [18].

Immunotherapy is used not only as a standalone treatment but also in combination with chemotherapy and radiotherapy and plays a crucial role as an adjunct to surgical treatment. Numerous studies evaluate the effectiveness of immune checkpoint inhibitors (ICI) therapy in combination with surgery, both as neoadjuvant (preoperative) and adjuvant (postoperative) therapies.

Another interesting study is IMvoke010. Here the recruitment process (400 patients) has been completed and results are awaited. The research includes patients with stage III HPV-positive HNSCC and stage IVa or IVb HPV-negative HNSCC. The participants will be randomly assigned to groups receiving atezolizumab or placebo after local treatment, including surgical resection. It is planned to provide the evaluation of OS and event-free survival.

Following the success of ICI therapy in R/M HNSCC, there has been growing interest in incorporating immunotherapy into radiotherapy or chemoradiotherapy for locally advanced HNSCC. The JAVELIN Head and Neck 100 phase III trial uncovered potential benefits of concurrent use of avelumab with chemoradiotherapy in patients with locally advanced HNSCC whose tumors exhibited high PD-L1 levels (>25%) [19].

In summary, the above studies suggest that patients with PD-L1 positive HNSCC benefit more from using immune checkpoint inhibitors than those with PD-L1 negative status.

### 2.2. CTLA-4

CTLA-4, a protein on the surface of activated T lymphocytes, limits the immune response. Although antibodies targeting CTLA-4 have not yet been officially approved as treatment for HNSCC patients, promising study results indicate their efficacy when combined with other checkpoint blockade methods [20].

Detailed analysis of the relationship between DNA methylation of CTLA-4 and response to ICI therapy has shown a correlation not only in immunotherapy efficacy but also in progression-free survival. Abnormal methylation of the CTLA-4 gene may be a predictive indicator of immunotherapy effectiveness in HNSCC patients [20,21].

Previous studies demonstrated that combinations of different immunotherapeutic antibodies are more effective than using single antibodies. In an innovative study using a preclinical HPV-positive oral cancer model (mEER) made of mouse epithelial cells from the tonsils, various immunotherapy strategies were tested using α-PD-1, α-CTLA-4, and α-Lag-3 antibodies. It was concluded that the combination of α-PD-1 and α-CTLA-4 therapy led to a better mouse survival and reduced tumor size compared to monotherapy or the combination of α-PD-1 and α-Lag-3. Both α-CTLA-4 and α-PD-1 monotherapies were effective, while α-Lag-3 monotherapy was less effective. The α-PD-1 and α-CTLA-4 combination positively influenced the proportions of immunosuppressive cells, improving the immune response. The combined α-PD-1 and α-CTLA-4 therapy was safe and showed no toxicity in trials.

The article highlights that higher levels of CTLA-4 were detected on CD8+ T cells in mEER tongue tumors, suggesting that CTLA-4 expression may be associated with a better response to α-PD-1 therapy. The study results showed that combining α-PD-1 with α-CTLA-4 significantly improved tumor-free survival compared to monotherapy. This suggests that CTLA-4 levels affect the efficacy of α-PD-1 therapy, and its blockade may be necessary for optimal results [22].

Dual blockade of immune checkpoints is increasingly gaining in importance as a new therapeutic strategy for patients in advanced stages of the disease. In the phase III Checkmate-067 clinical trial, higher median progression-free survival was observed in patients treated with combined nivolumab and ipilimumab therapy compared to monotherapy (ipilimumab or nivolumab)-PFS: 11.5 months for combined therapy compared to 2.9 and 6.9 months for monotherapy [23]. The previously mentioned phase III CheckMate 651 trial also confirmed the beneficial therapeutic effect of nivolumab and ipilimumab therapy in extending OS and achieving durable responses in patients exhibiting the PD-L1 biomarker [18].

It is worth noting that combining CTLA-4 inhibitors with PD-L1 inhibitors can significantly enhance treatment efficacy through synergistic action for activating T lymphocyte. Despite many studies indicating elevated CTLA-4 expression in HNSCC, the effectiveness of CTLA-4 therapy remains a subject of discussion [24].

In summary, CTLA-4 is an important therapeutic target, and its blockade may represent a valuable strategy in treating certain cancers, such as HNSCC, especially when used in conjunction with other immunotherapy methods [20].

### 2.3. HPV

One of the main etiological factors for the occurrence of HNSCC, right after smoking and alcohol consumption, is a viral infection, most often with the human papillomavirus (HPV). In the population of alcohol consumers, habitual smokers, and carriers of the HPV virus, there is a synergistic increase in the risk of developing head and neck squamous cell carcinoma. Depending on the location of the tumor in the oral cavity, it is estimated that HPV is present in 25% of HNSCC cases. Among HPV-positive head and neck squamous cell carcinomas, most cases are associated with the presence of HPV-16, accounting for 90% of occurrences. Until now, it has been suggested that HPV-positive and HPV-negative tumors show fundamental molecular differences. However, despite numerous studies on therapies based on HPV status, conducted both at the clinical and preclinical levels, there are still no definitive guidelines for treating patients with HNSCC depending on HPV status [25,26]. It is postulated that HPV-positive HNSCC shows better prognosis and more effective response to ICI treatment due to the presence of a less immunosuppressive environment [27,28].

The role of HPV status in HNSCC as a predictive biomarker in immunotherapy treatment has not yet been clearly defined. In the KEYNOTE-012 and KEYNOTE-055 studies evaluating the efficacy of pembrolizumab response in HNSCC patients, response to therapy was observed regardless of HPV virus status [28]. Therefore, it can be argued that PD-L1 expression is independent of the tumor’s HPV status. However, higher CTLA-4 expression has been demonstrated in HPV-positive tumors, suggesting its role as a trigger for activating the immune response and host defense mechanisms against cancer cells [29].

Another study where no differences in ICI therapy efficacy depending on the tumor’s HPV status were found is the CheckMate141 study. However, researchers emphasize that the group of patients where the risk of death decreased by 61% constituted PD-L1 and HPV-positive tumors [16,17].

Different conclusions regarding HPV status were observed in a retrospective review of 54 patients treated with pembrolizumab or nivolumab at the Moffitt Cancer Center between 2016 and 2018, where it was shown that patients with HPV-positive HNSCC achieved a higher OS of 13.5 months compared to HPV-negative patients, and a longer duration of ICI administration (7 months compared to 3 months). The above data show that HPV status is associated with a better response to ICI therapy and its undeniable role as a predictive factor in HNSCC treatment [30,31,32].

Another study showing the difference in ICI therapy for HPV-positive and HPV-negative patients is the phase II HAWK study, which evaluated the efficacy of durvalumab and anti-PD-L1 monoclonal antibody treatment in PD-L1-positive patients with R/M HNSCC. A higher response rate to treatment was noted in patients with HPV-positive tumors compared to patients with HPV-negative tumors (18.6% higher in HPV-positive patients), as well as a higher median overall survival rate in patients with HPV-positive tumors than in HPV-negative patients (10.2 months compared to 5 months) [33].

Despite many discrepancies in the data, it can be confidently stated that both HPV-positive and HPVnegative patients benefit from HNSCC checkpoint inhibitor treatment. Both groups of patients may exhibit similar levels of immune cells (such as T-regs and CD56 NK cells), but their tumor microenvironment shows some discrepancies, such as higher expression of CD8+ TIL. Although the mechanisms of response to the therapy are different, strategies aimed at manipulating the immune system may be effective in both patient groups. Further studies on the molecular and immunological profiles of these cancers may help understand these differences better and tailor the therapy to the individual needs of patients [31].

### 2.4. p53 and Apoptosis

The p53 protein, which is encoded by the TP53 gene and exhibits tumor suppressor properties, is involved in the proper regulation of the cell cycle and is a key element of the innate immune response. When DNA damage or cellular stress is caused by, for example, oxygen deficiency, the p53 protein is activated and induces cell apoptosis. A characteristic feature of cancer cells in HNSCC is resistance to death by avoiding apoptosis. The most common mutation is the one in TP53, which has been the subject of many studies [34,35,36].

There are three main mechanisms of change in the TP53 gene: somatic mutation and degradation utilizing E6 or MDM2. TP53 gene mutation, occurring in half of HNSCC cases, affects the composition of immune cells in the tumor. In the analysis of HNSCC patients, samples with TP53 mutation contained fewer specific types of immune cells (B cells, CD8+ T cells, and NK cells), which weakens the immune response against the tumor. At the same time, activation of JAK-STAT signaling in these cancers with TP53 mutation leads to an increase in other immune cells (neutrophils, macrophages, and CD4 cells), suggesting that TP53 mutation alters the tumor microenvironment and may affect its development and response to therapy [35].

Studies suggest that p53, especially in its mutated form, can be effectively utilized in cancer immunotherapy because it is recognized by the immune system and triggers immune responses. An interesting feature is the ability of p53 to regulate PD-L1 expression through miR34 [37].

It is currently believed that a higher percentage of tumor mutations, including TP53, indicates a worse clinical course for HNSCC patients [37,38]. Deactivation of p53 leads to the accumulation and burdening of additional mutations in cancer cells, reducing their sensitivity to radio or chemotherapy. The predictive value in the context of ICI treatment response and tumor mutational burden (TMB), including TP53 mutations, has been confirmed in HNSCC for pembrolizumab and durvalumab [38]. It is worth noting the significantly increased infiltration of macrophages, CD8+, and CD4+ cells in HNSCC, which is crucial information for developing new therapies for HNSCC [37].

So far, several drugs targeting p53 have proven effective, and the value of Gendicine gene therapy in combination with radiotherapy accompanied by Advexin as an intratumoral injection has been confirmed [39]. Additionally, the clinical potential of immunotherapy based on genetically modified T cells targeting tumors with mutated TP53 is emphasized [40].

Gricerol and PRIMA-1 can induce apoptosis of cancer cells in HNSCC by acting on the mutated form of TP53, while nutlin-3 and RITA are successfully used to block TP53 by MDM-2. Both treatment methods additionally enhance the efficacy of chemo- and radiotherapy [32,39].

It is worth examining the relationship between TP53 mutations and human papillomavirus (HPV) infection in HNSCC patients. HPV-negative HNSCCs are more likely to present TP53 mutations, leading to the loss of its tumor-suppressor function, while in HPV-positive cancers, such mutations are rarer or absent [41]. In HPV-positive tumors, despite low levels of wild-type p53 degraded by viral E6 proteins, apoptosis can occur, whereas in HPV-negative cancers, p53 is typically mutated and inactivated [42].

In HPV-negative HNSCCs, mutated p53 can contribute to greater resistance to treatment due to inhibited cell apoptosis, autophagy, multidrug resistance, and promotion of stem cell properties in cancer cells [41]. In the study evaluating the efficacy of the proteasome inhibitor bortezomib to prevent p53 degradation and restore its function in HPV-related HNSCC cells, a favorable response was observed in HPV-positive tumors. Unlike HPV-negative HNSCCs, HPV-positive HNSCCs exhibited restoration of p53 function, cell cycle arrest in the G1 phase, and induction of apoptosis. Although restoration of p53 function with bortezomib does not significantly increase sensitivity to conventional therapies (chemo- and radiotherapy) in HPV-positive HNSCC cells, it may potentially increase their immunogenicity and improve the efficacy of immunotherapy. Strategies aimed at stabilizing p53, thereby inducing apoptosis and triggering tumor antigen release, may prove beneficial in immunotherapy [42].

## 3. Discussion

Owing to the successful development of immunotherapy, cancer treatment is becoming increasingly effective and targeted. Currently, a rapidly advancing field of research is targeting specific receptors—targeted therapy in oncological treatment. One of these is uPAR, associated with aggressive tumor features, invasion, and lymph node metastasis, which has been utilized in nanomedicine—targeted CRDA nanoparticles aimed at the uPAR receptor. Upon reaching the uPAR receptors, which are overexpressed in tumor cells, their elimination is achieved through irradiation with near-infrared lasers and ultrasound [43,44]. Targeted therapies against EGFR, whose levels significantly increase in tumor cells but are also found in healthy host tissues, show limited effectiveness. A newly developed type of nanoparticle targeting EGFR exclusively in an acidic environment may solve the issue of the receptor’s lack of tumor specificity [43]. Precision in treatment could be enhanced by studying the genome of HNSCC patients and identifying mutated TP53 neoantigens as targets for precise therapy [37]. The complexity of head and neck squamous cell carcinoma biology and unpredictable drug resistance still pose challenging obstacles in treating the disease. A promising direction is the adjustment of HNSCC therapy based on dynamic changes occurring in the tumor microenvironment, including changes in the neutrophil-to-lymphocyte ratio, fluctuations in CRP values, and microsatellite instability. The implementation of tools such as liquid biopsy would increase the precision of therapy selection. It is worth mentioning that treatment with immune checkpoint inhibitors (ICIs) modifies the tumor microenvironment, making subsequent administration of traditional drugs, such as cisplatin or 5-FU, enhance the tumor’s sensitivity to therapy [45]. An attractive treatment strategy also involves targeting extracellular matrix (ECM) signaling pathways, such as the TGF-beta pathway or the CD44 receptor. Fluorothiazinon (FT), Flumatinib (HF), Bintrafusp alfa, and Dalantercept, which affect the TGF-beta pathway, demonstrate promising clinical effects [46]. It seems that aiming at several independent therapeutic targets simultaneously would bring greater clinical benefits. Evidence for the effectiveness of therapies targeting multiple checkpoints comes from the results of the phase III clinical trials CheckMate-067 and CheckMate-651, in which the effects of nivolumab and ipilimumab were utilized. Future treatment directions for HNSCC should focus on tailoring specific therapies to the individual needs of each patient based on molecular analysis of cancer cells using the NGS method [39]. The use of immunotherapy in HNSCC treatment yields promising results. However, its still-insufficient effectiveness underscores the need to search for new biomarkers and select patient groups who will benefit most from the therapy. It is important to mention the occurrence of toxic effects resulting from ICI therapy, particularly with pembrolizumab and nivolumab, in organs that also express PD-L1, such as lungs. Moreover, not all patients respond to this type of treatment, which highlights additional limitations of this therapy. Further research is necessary to fully utilize the potential of immunotherapy in this group. Therapeutic options for patients with recurrent/metastatic (R/M) HNSCC remain severely limited. Up to 40–50% of patients with locally advanced squamous cell carcinoma of the oral cavity experience recurrence despite the use of multimodal treatment methods. In classic chemotherapy using cetuximab with cisplatin and fluorouracil, a response rate of only 36% was achieved, with a PFS of 5.6 months. Additionally, radio- and/or chemotherapy treatments lead to many significant side effects, greatly reducing the patients’ quality of life. Despite the large-scale expansion of research on HNSCC treatment strategies, there still is a critical need to identify targeted biomarkers for immunotherapy [1,3,43,47].
